# Supplementary figures and images for: Nonparametric Limits of Agreement in Method Comparison Studies: A Simulation Study on Extreme Quantile Estimation
Source: Int J Environ Res Public Health. 2020 Nov 11;17(22):8330. doi: 10.3390/ijerph17228330 (PMC7698333; doi:10.3390/ijerph17228330)

**NO, n=250**

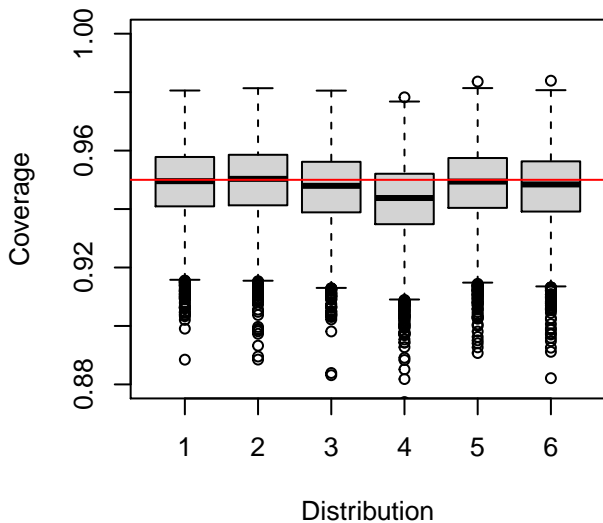

**NO, n=500**

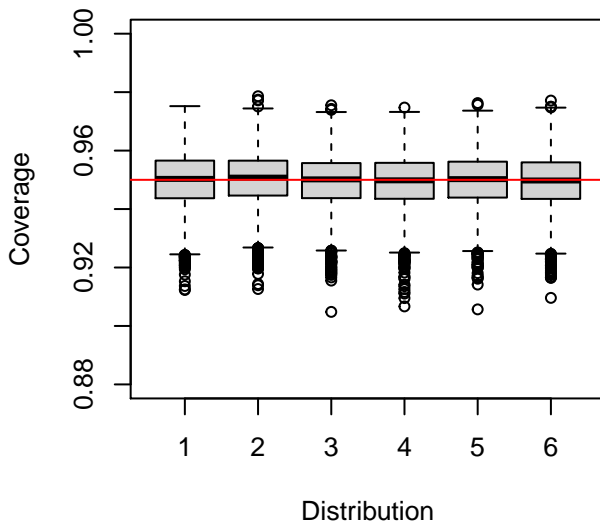

**NO, n=750**

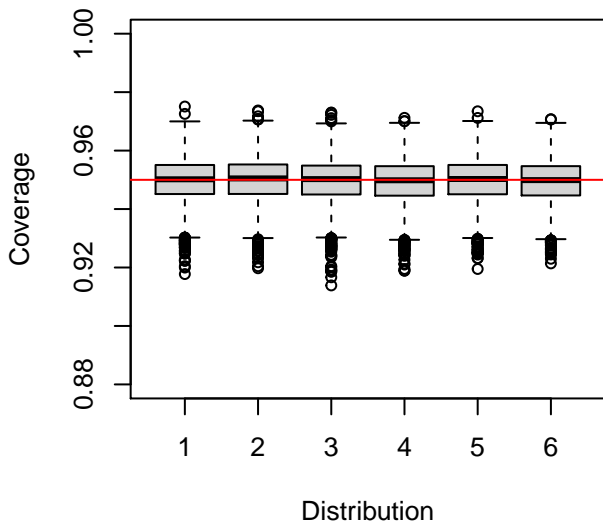

**NO, n=1000**

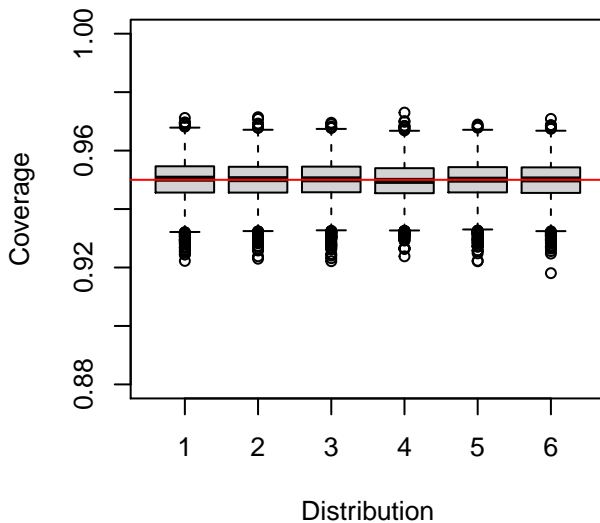

Supplement: Supplementary file 1 [file ijerph-17-08330-s001.zip › Figure S10 NO - 250-1000.pdf]

**SQ, n=250**

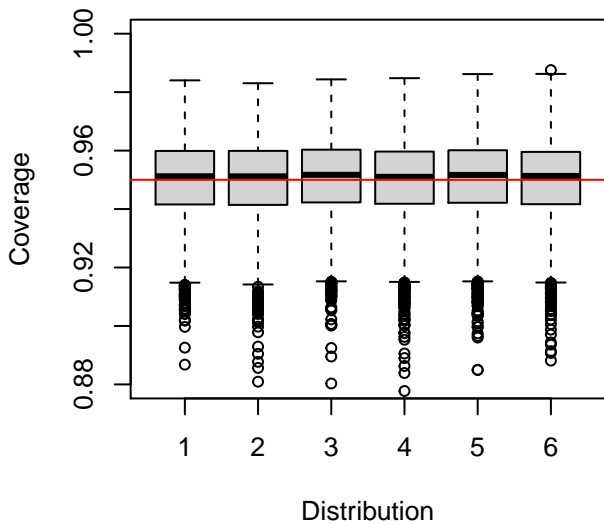

**SQ, n=500**

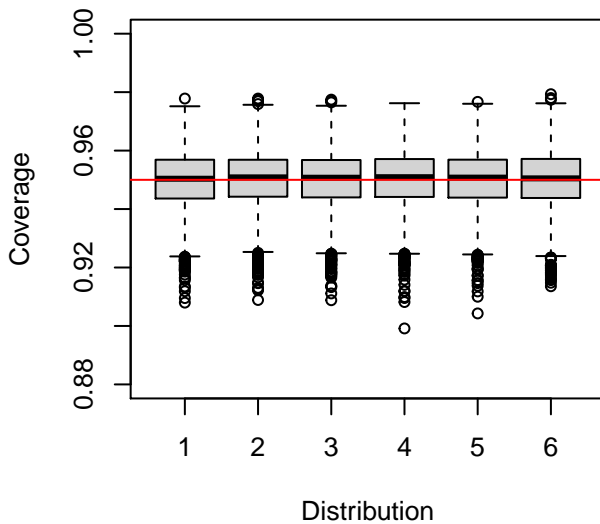

**SQ, n=750**

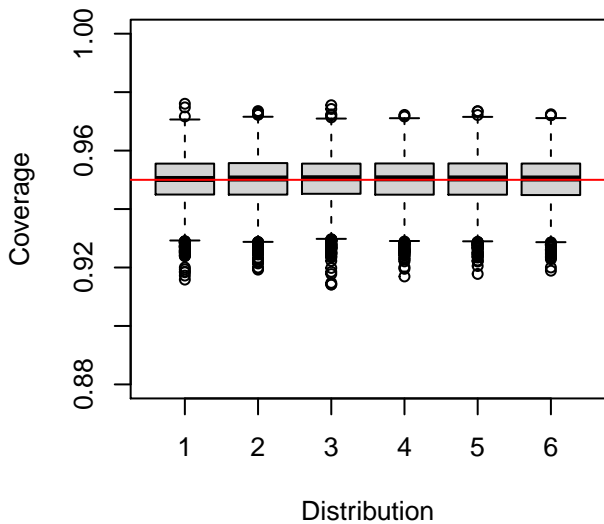

**SQ, n=1000**

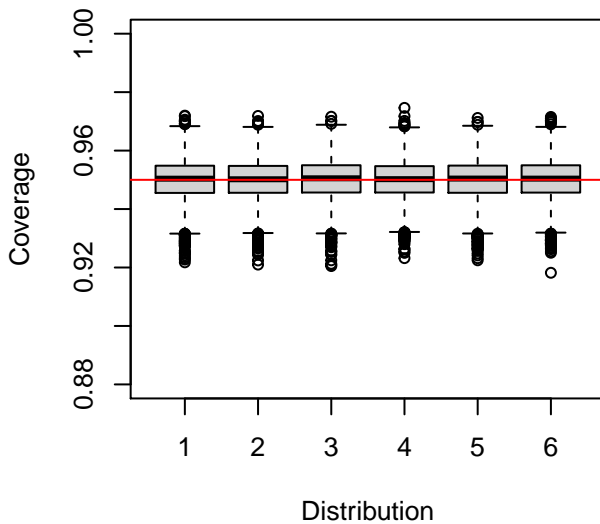

Supplement: Supplementary file 1 [file ijerph-17-08330-s001.zip › Figure S2 SQ - 250-1000.pdf]

**HD, n=250**

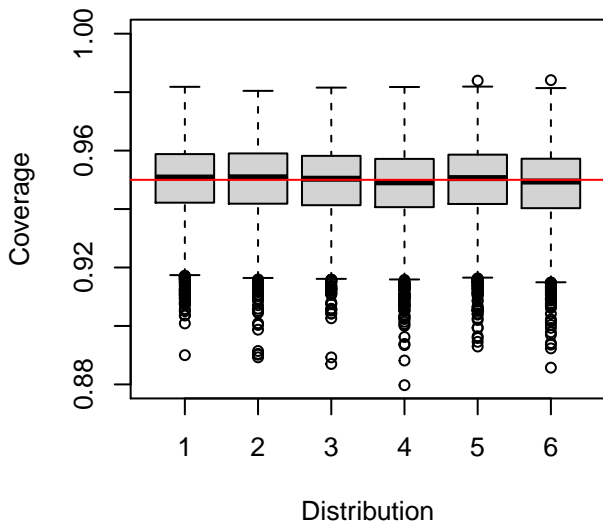

**HD, n=500**

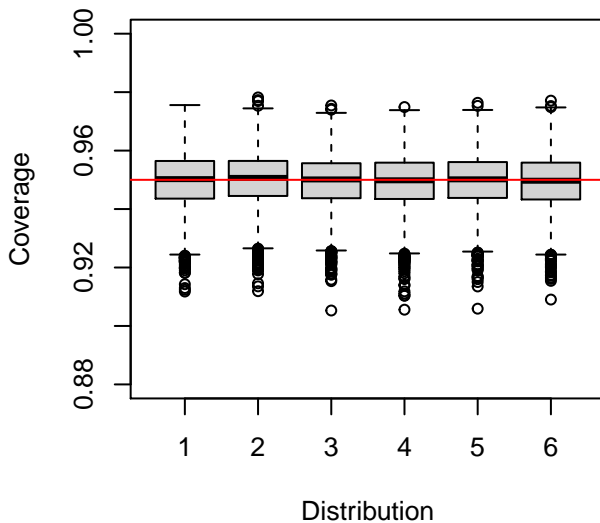

**HD, n=750**

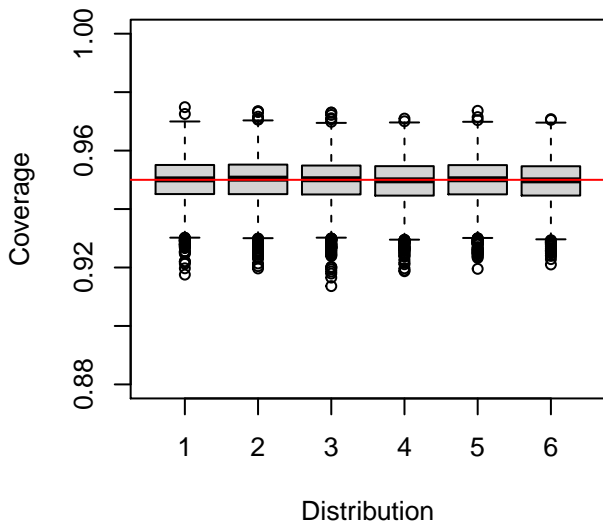

**HD, n=1000**

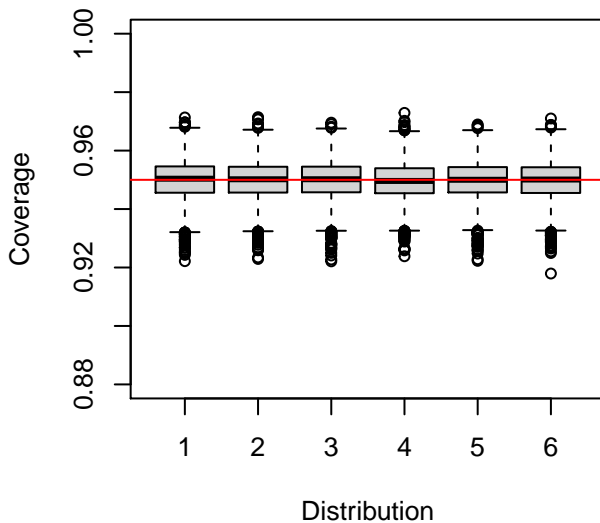

Supplement: Supplementary file 1 [file ijerph-17-08330-s001.zip › Figure S3 HD - 250-1000.pdf]

**BP, n=50**

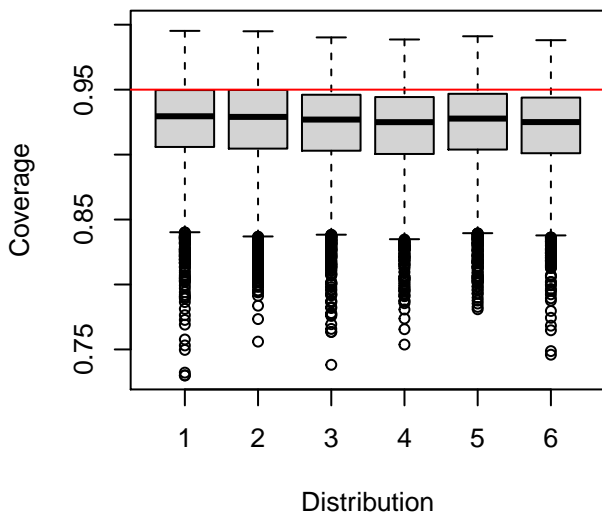

**BP, n=100**

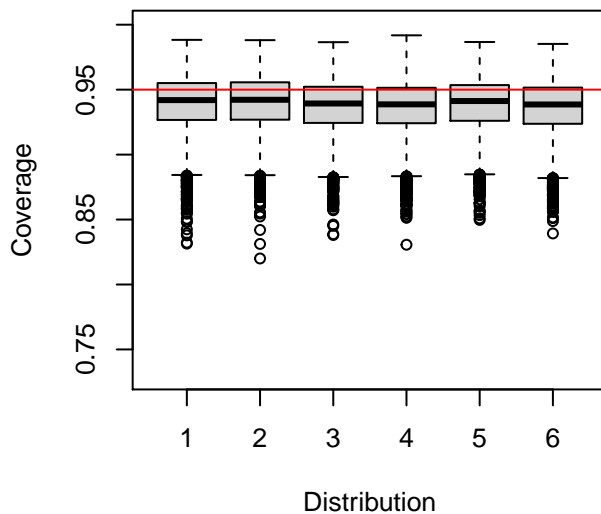

**BP, n=150**

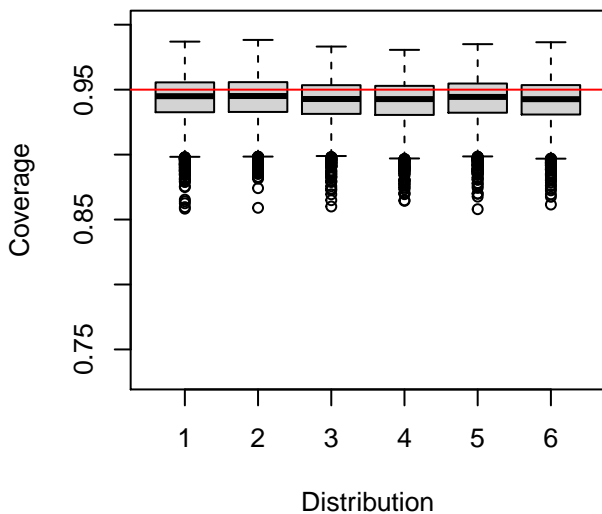

**BP, n=200**

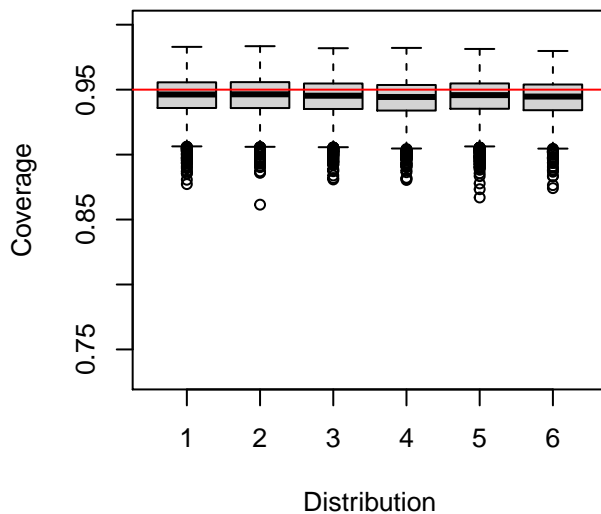

Supplement: Supplementary file 1 [file ijerph-17-08330-s001.zip › Figure S4 BP - 50-200.pdf]

**BP, n=250**

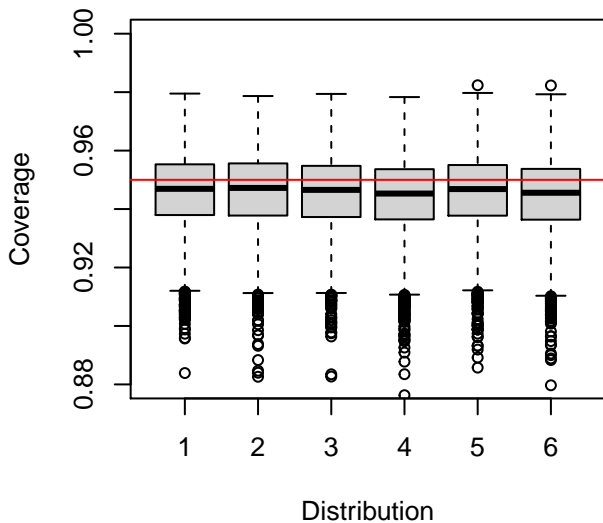

**BP, n=500**

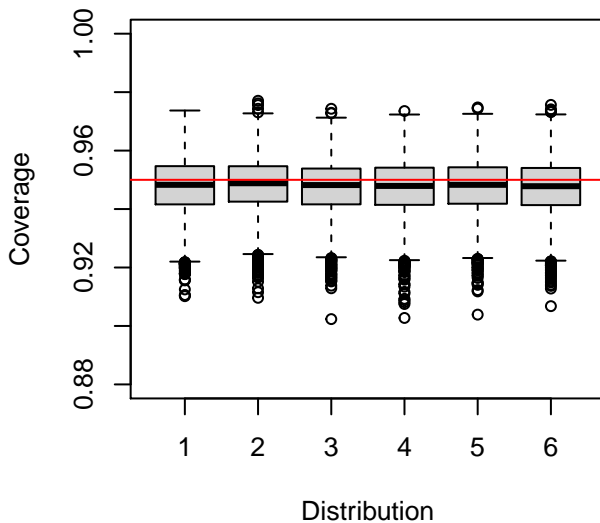

**BP, n=750**

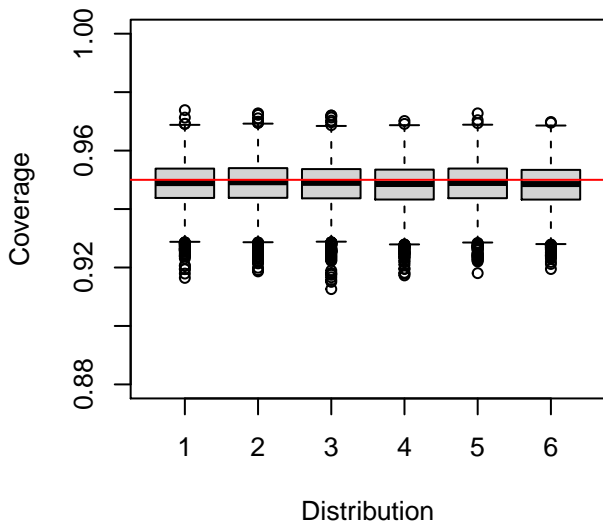

**BP, n=1000**

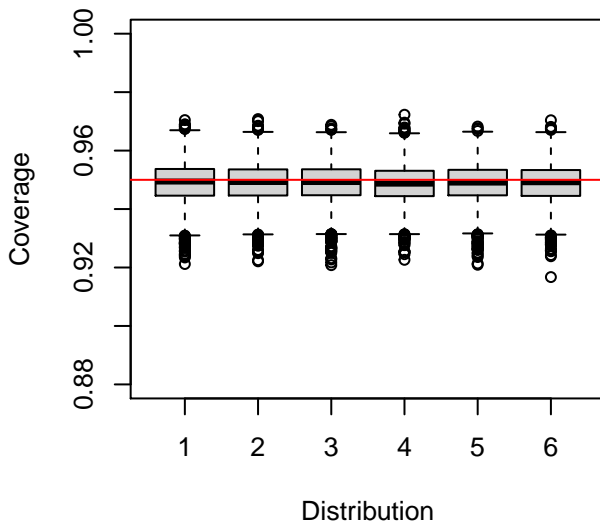

Supplement: Supplementary file 1 [file ijerph-17-08330-s001.zip › Figure S5 BP - 250-1000.pdf]

**HD lc, n=50**

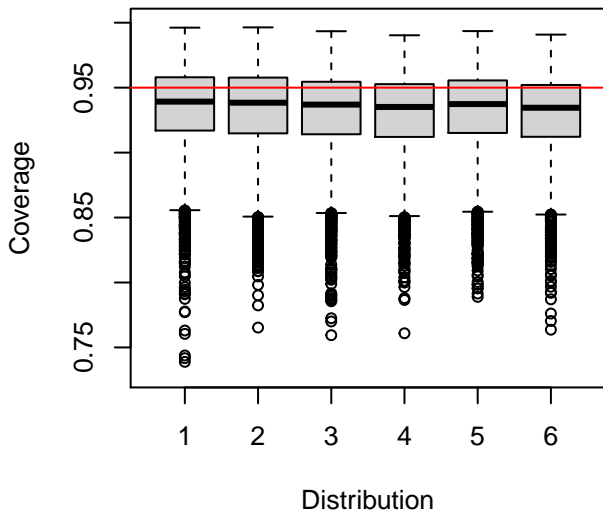

**HD lc, n=100**

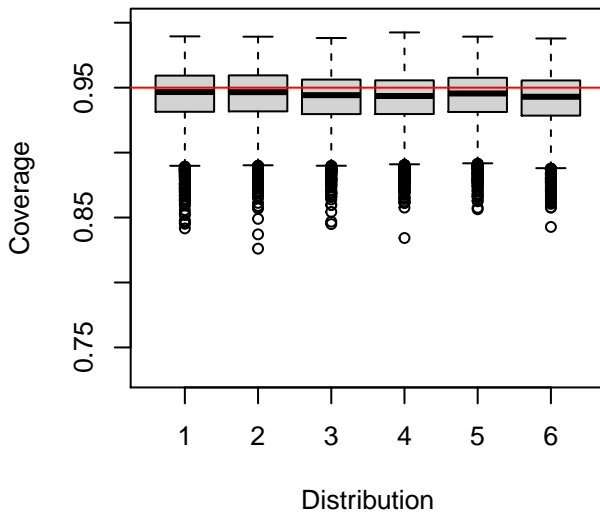

**HD lc, n=150**

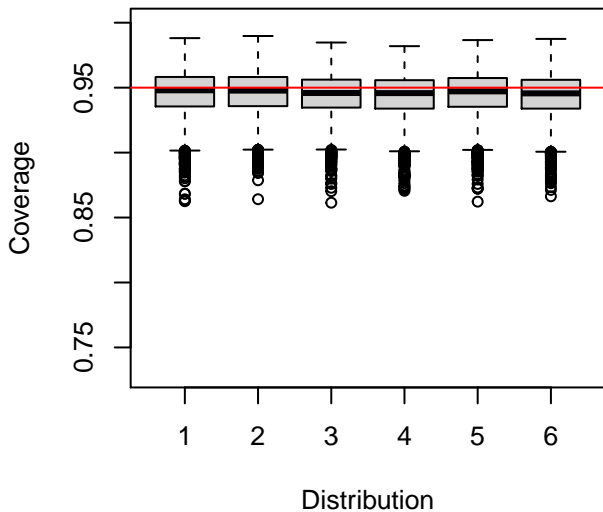

**HD lc, n=200**

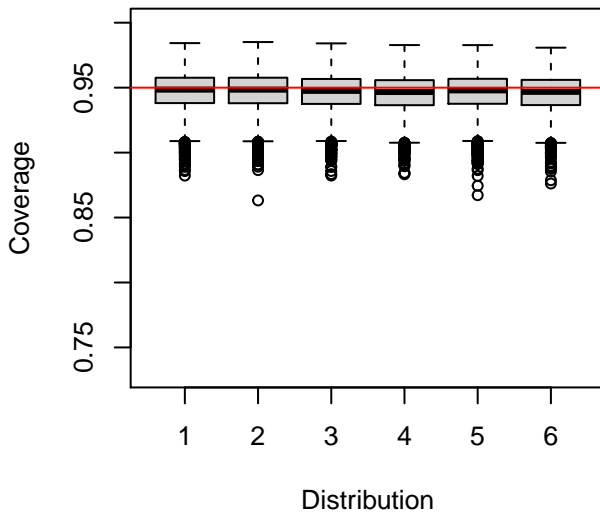

Supplement: Supplementary file 1 [file ijerph-17-08330-s001.zip › Figure S6 HD lc - 50-200.pdf]

**HD lc, n=250**

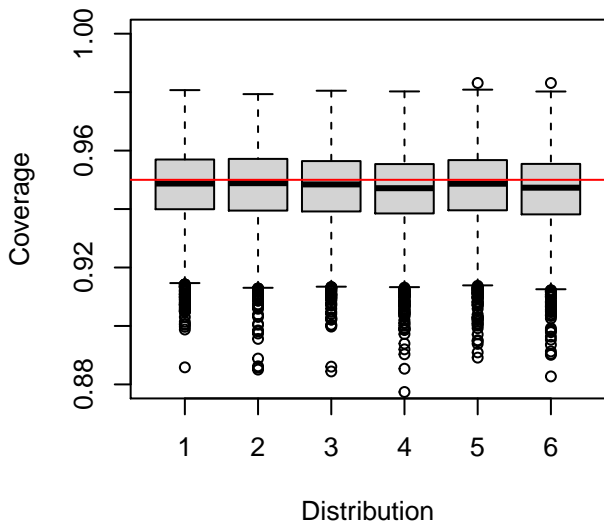

**HD lc, n=500**

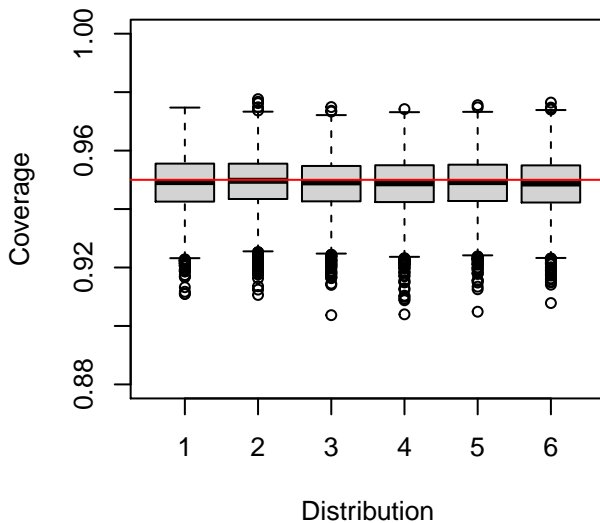

**HD lc, n=750**

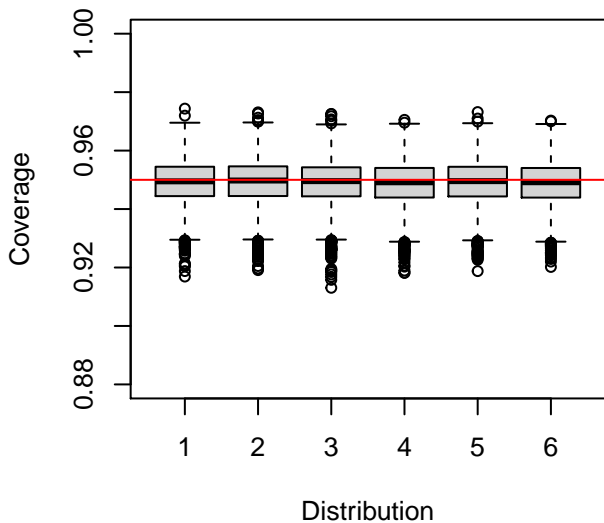

**HD lc, n=1000**

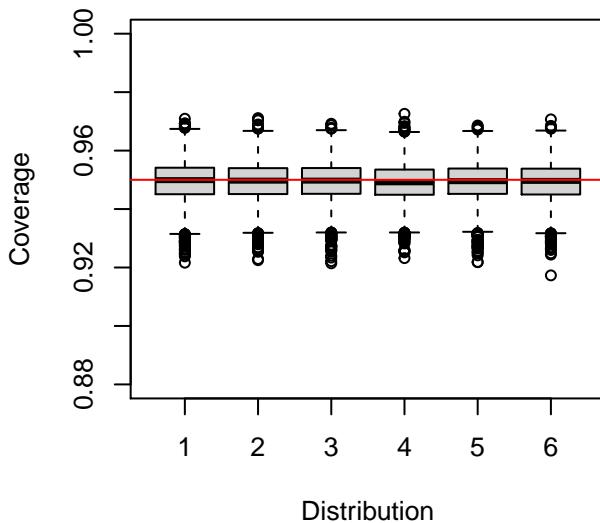

Supplement: Supplementary file 1 [file ijerph-17-08330-s001.zip › Figure S7 HD lc - 250-1000.pdf]

**SV, n=250**

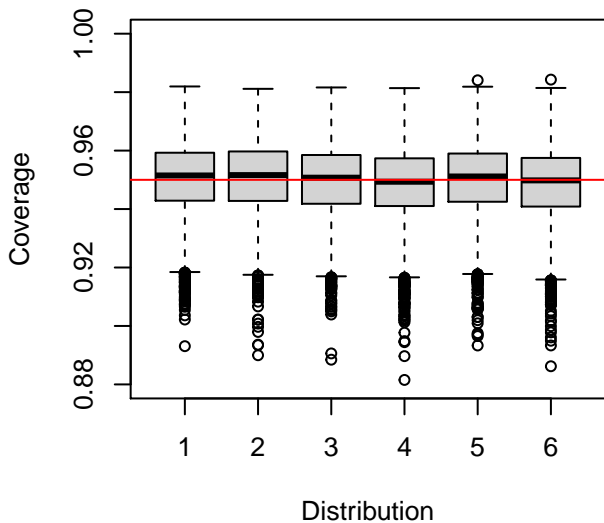

**SV, n=500**

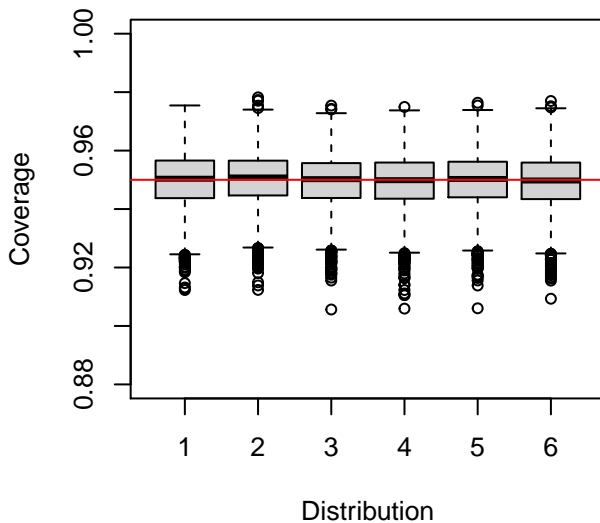

**SV, n=750**

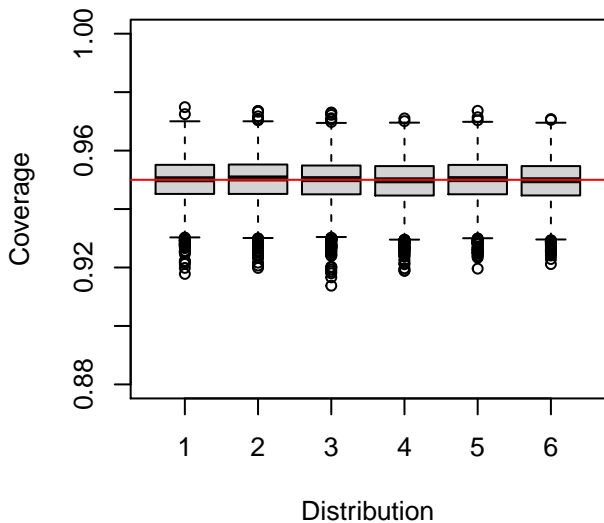

**SV, n=1000**

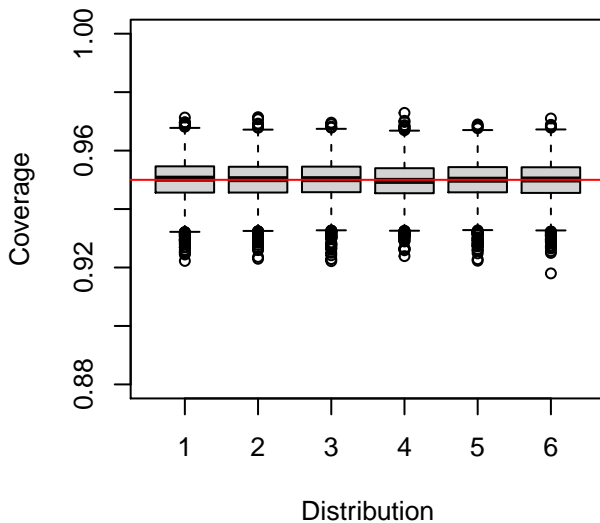

Supplement: Supplementary file 1 [file ijerph-17-08330-s001.zip › Figure S8 SV - 250-1000.pdf]

**NO, n=50**

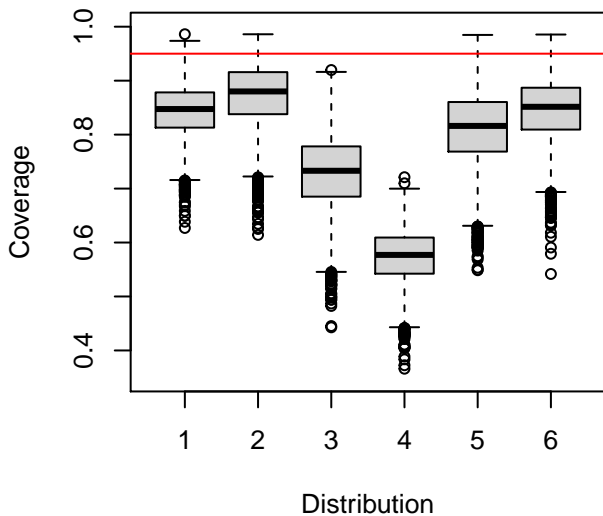

**NO, n=100**

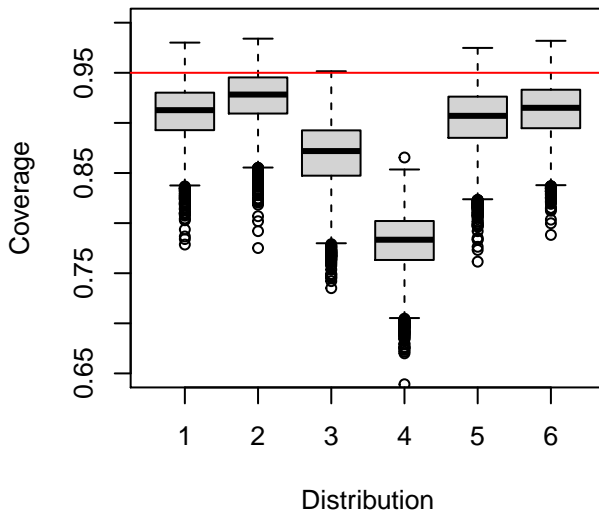

**NO, n=150**

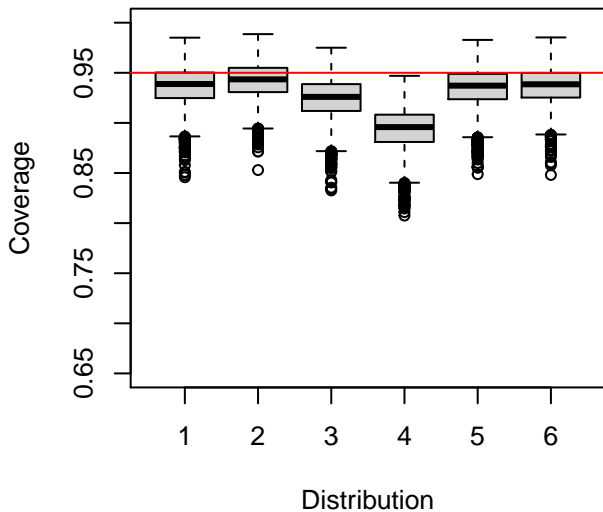

**NO, n=200**

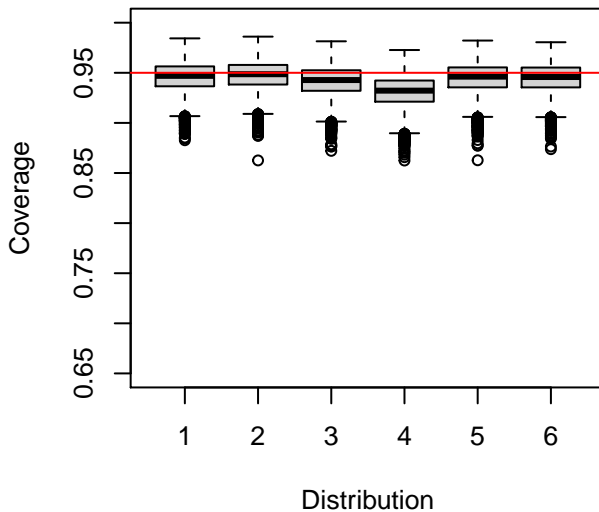

Supplement: Supplementary file 1 [file ijerph-17-08330-s001.zip › Figure S9 NO - 50-200.pdf]
